# Genetic diversity and population structure analysis of Indian blackberry (*Syzygium cumini* L.) using CAAT box‑derived polymorphism (CBDP) and start codon targeted polymorphism (SCoT) markers

**DOI:** 10.1016/j.jgeb.2025.100468

**Published:** 2025-02-13

**Authors:** Ajay Kumar, Kanhaiya Singh, Amit Kumar Singh, Jai Prakash, Amit Kumar Goswami, Gyan Prakash Mishra, Vishaw Bandhu Patel, Suman Lata, Anshuman Singh

**Affiliations:** a The Graduate School, ICAR-Indian Agricultural Research Institute, New Delhi 110012, India; b Division of Fruits and Horticultural Technology, ICAR-Indian Agricultural Research Institute, New Delhi 110012, India; c ICAR-National Bureau of Plant Genetic Resources, New Delhi 110012, India; d Division of Genetics, ICAR-Indian Agricultural Research Institute, New Delhi 110012, India; e Horticultural Science Division, ICAR-Krishi Anusandhan Bhawan-II, New Delhi 110012, India; f Division of Vegetable Science, ICAR-Indian Agricultural Research Institute, New Delhi 110012, India; g ICAR- Central Institute for Subtropical Horticulture, Lucknow 226101, India

**Keywords:** AMOVA, CBDP, Genetic diversity, Gene-targeted markers, Population structure, SCoT, *Syzygium cumini* (L.)

## Abstract

Indian blackberry (*Syzygium cumini* L.) also known as jamun is a very important underutilized fruit crop with notable medicinal and economic value. However, its genetic improvement has been constrained by limited knowledge of the genetic diversity within existing collections. Therefore, a comprehensive characterization of genetic diversity in this species, using molecular tools, is essential to support effective germplasm management and application in breeding programs. In this investigation, a total of 32 jamun genotypes consisting of 30 seedling-origin genotypes, one improved cultivar CISH J-37 and one wild genotype (*Syzygium fruitecosum*) were analysed using the two gene-targeted markers, CBDP and SCoT. In total, 29 primers (22 CBDP and 7 SCoT primers) detected genetic polymorphism across the genotypes. The CBDP markers amplified a higher polymorphism percentage, 94.85% across 291 bands, than the SCoT markers, 92.75% across 69 bands. The mean PIC values for CBDP and SCoT were 0.28 and 0.31, respectively. MI values were higher for CBDP (3.21) than for SCoT (2.88). Cluster analysis using UPGMA identified six clades, which grouped genotypes into seedling-origin, improved and wild categories. The PCoA based on molecular profiling data of CBDP, SCoT and both together explained 26.65%, 38.39% and 23.22% of the variation respectively. AMOVA results revealed that 85–90% of genetic variation existed within populations. Bayesian STRUCTURE analysis grouped genotypes into two major populations confirming genetic divergence between seedling-origin, improved and wild genotypes. This study is the first to integrate CBDP and SCoT markers for genetic diversity analysis of the Indian blackberry. The results highlight the utility of these markers in genetic variation assessment and would help design germplasm conservation and breeding strategies in this crop.

## 1 Introduction

Indian blackberry (*Syzygium cumini* L.) is an underutilized fruit crop of the Myrtaceae family. It is known globally by various names, such as jamun, jambul, danson plum, jambolana, and black plum.[1], [2] It is native to India and has a wide distribution across the Indian subcontinent and many regions of South Asia, including India, Burma (Myanmar), Nepal, Sri Lanka, Bangladesh and Pakistan.^3^ Additionally, it has been successfully domesticated in numerous countries across Asia, America and Africa which shows its adaptability and horticultural significance.^4^ As one of the hardiest fruit crops, it can be easily cultivated in marshy and neglected fields where other fruit plants are difficult to grow commercially.^5^ The purplish berry-like fruits contain a single seed and are consumed as fresh and processed into various products like juice, jam, jelly, vinegar, wine, pudding ice cream and jamun strips.[6], [7] The fruits, seeds, bark and leaves of this crop are traditionally utilized in Ayurvedic, Unani and Chinese medicinal systems as a natural treatment for hyperglycemia, dysentery, glycosuria, asthma, bronchitis and ulcers.^8^ Notably, it has been found as an effective remedy for diabetes.^9^ The plant's various parts possess significant health activities including anti-anemic, antioxidant, antibacterial, antiallergic, hypolipidemic, hepatoprotective and antipyretic.^10^ Due to their potential for use as both food and healthcare, as well as their outstanding biological activities, the genus *Syzygium* is recognized for its economic significance.^11^

Leveraging genetic diversity and understanding the relationships within crop germplasm collections is essential for their effective management, application in breeding programs, and enhancing the success of breeding efforts.[12], [13], [14] A greater range of molecular markers are utilized to detect diversity at the genetic level across different plant species. In underutilized crops, genetic diversity studies have primarily employed non-locus specific markers, such as RAPD and ISSR which have several disadvantages such as low reproducibility, non-specific binding, lower genetic resolution and limited insight into trait association. To overcome these challenges many novel marker techniques were developed which unravel high polymorphism from the genic or functional region of the genome and thus could provide more comprehensive information on genomic level genetic diversity present in crop germplasm collections.[15], [16], [17], [18] Two key gene-based markers CAAT box-derived polymorphism (CBDP) and start codon targeted (SCoT) are widely utilized in genetic diversity studies of crops. Of these two, CBDP was developed by our team and utilizes promoter-targeted primers to take advantage of the CAAT box consensus sequence within promoters of plant genes.^19^ On the other hand, SCoT markers emerge as another promising technique that draws from the conserved regions of DNA adjacent to the ATG start codon within the genes of plants. Hence, it can capture genetic differences within a particular gene linked to specific traits.^20^ These techniques are gaining popularity for molecular marker-based studies in crops due to their cost-effectiveness, high polymorphism and reproducibility over traditional dominant markers. Additionally, since genomic sequence information is not prerequisite for primer designing, these markers can be applied to any crop species.[19], [21], [22] The CBDP marker technique was first time validated in jute, linseed and cotton cultivars.^19^ Subsequently, it has been utilized for genetic diversity and gene mapping studies in many crop species.[23], [24], [25] Additionally, SCoT markers have proven effective in assessing genetic diversity, DNA fingerprinting and determining population structure in several horticultural plant species.[26], [27], [28], [29], [30], [31], [32]

In recent years many studies have combined these CBDP and SCoT gene-targeted markers for generating comprehensive insights into genetic diversity and population structure in different plant species including *Simmondsia chinensis,*^33^ *Andrographis paniculata,*^34^ *Triticum durum,*^16^ *Prosopis cineraria,*^35^ *Bauhinia racemosa* Lam.,^36^ *Clerodendrum serratum* L.,^37^ *Aegilops triuncialis,*^38^ *Triticum aestivum* L.,^39^ *Mangifera indica* L.,^40^ *Hordeum vulgare* L.,^41^ *Cannabis sativa* L.,^42^ *Centaurea species,*^43^ *Abelmoschus esculentus* L.,^44^ and *Senna alexandrina* Mill..^45^ These markers efficiently determined genetic relationships, clustering and population structures.[39], [42] Their combined application demonstrated comprehensive patterns of genetic diversity, making them more valuable than single markers application in the study of crop diversity.^40^ Positive markers correlations have been found reliable and accurate for the analysis of genetic variability.^37^ SCoT and CBDP markers were effective tools for evaluating variability and relatedness, even under complex classification conditions.^43^

Despite advances in molecular marker technology, their use in underutilized crops like jamun remains limited, creating a huge knowledge gap in the genetic diversity of this crop. Previous studies have mainly used RAPD,[46], [47], [48] ISSR[49], [50] and RAPD + ISSR.[51], [52], [53], [54] To date, just one report has utilized CBDP markers to explore genetic diversity in this crop.^55^ This necessitates characterizing the genetic diversity of existing jamun collections with different sets of reliable markers such as gene-targeted markers. In this context, we hypothesize that the combination of CAAT box-derived polymorphism (CBDP) and start codon targeted (SCoT) polymorphism markers will significantly enhance the detection of genetic diversity and population structure in jamun collections. Therefore, in the current study, we first time combined both gene-targeted markers (CBDP and SCoT) to evaluate their effectiveness in assessing genetic diversity and a comprehensive understanding of the genetic relationships among jamun genotypes.

## 2 Material and methods

### 2.1 Sampling

**Fig. 1:**
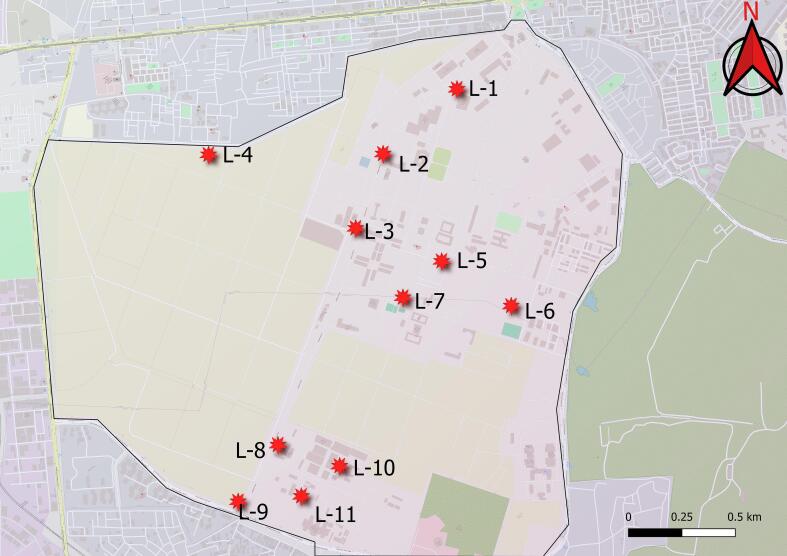
Map illustrating jamun sample collection sites.

The study was conducted on a large natural population of jamun spread across the Pusa campus, New Delhi, situated in the semi-arid tropics of India. A total of 31 genotypes have been selected from different 11 sites (Fig. 1). Besides, one improved genotype namely CISH J-37 (Jamwant) was also included in our study. Two to four samples were randomly obtained at each site, with a minimum distance of 20 m between all sampled individuals. Table 1 contains detailed information about the longitudes and latitudes of each genotype.

**Table 1:** Detailed information about the longitudes and latitudes of 32 Indian blackberry genotypes.

**Site**	**Genotype**	**Information**	**Latitude**	**longitude**
Location-1	PCJ-1	Seedling origin	28.64051010	77.16974820
	PCJ-2	Seedling origin	28.64079330	77.16818850
	PCJ-30	Seedling origin	28.64059880	77.16891190
Location-2	PCJ-3	Seedling origin	28.64089260	77.16689040
	PCJ-4	Seedling origin	28.64071590	77.16604070
	PCJ-5	Seedling origin	28.64070790	77.16543900
	PCJ-6	Seedling origin	28.64011710	77.16475400
Location-3	PCJ-7	Seedling origin	28.63910420	77.16301150
	PCJ-8	Seedling origin	28.63865840	77.16185110
	PCJ-12	Seedling origin	28.63762700	77.16108800
	PCJ-13	Seedling origin	28.63710300	77.16106800
Location-4	PCJ-9	Seedling origin	28.64495900	77.15882000
	PCJ-10	Seedling origin	28.64595600	77.15770400
Location-5	PCJ-11	Seedling origin	28.63717270	77.16309700
	PCJ-14	Seedling origin	28.63564700	77.16383240
	PCJ-15	Seedling origin	28.63530490	77.16373220
Location-6	PCJ-16	Seedling origin	28.63145900	77.16514300
	PCJ-17	Seedling origin	28.63242700	77.16281690
	PCJ-29	Seedling origin	28.63314600	77.16549200
Location-7	PCJ-18	Seedling origin	28.63516050	77.16122150
	PCJ-19	Seedling origin	28.63795560	77.16014060
Location-8	PCJ-20	Seedling origin	28.63401590	77.15229990
	PCJ-21	Seedling origin	28.63382300	77.15148900
	*S. fruiticosum*	Wild type	28.63448820	77.15331960
Location-9	PCJ-22	Seedling origin	28.63284790	77.14905820
	PCJ-23	Seedling origin	28.63190800	77.15232560
Location-10	PCJ-24	Seedling origin	28.63129140	77.15338500
	PCJ-25	Seedling origin	28.63131490	77.15401320
	PCJ-26	Seedling origin	28.63115300	77.15472030
Location-11	PCJ-27	Seedling origin	28.62994280	77.15314920
	PCJ-28	Seedling origin	28.63023110	77.15256030
	CISH-37	Cultivated type		

### 2.2 Extraction of genomic DNA

Fresh, disease-free leaves were explicitly collected to extract total genomic DNA from each sample following cetyltrimethylammonium bromide (CTAB) procedure by Doyle and Doyle,^56^ with some modifications. The main modification was the addition of 3 % of polyvinyl pyrrolidone (PVP) in the extraction buffer to remove phenolic content from leaf samples. The extracted DNA's integrity and purity were assessed using 1 % agarose gel and its concentration was measured through spectrophotometry. Finally, a working genomic DNA stock of each genotype was prepared with a final DNA concentration of 50 ng/µl.

### 2.3 PCR of CBDP markers

The study used a total of 40 CBDP primers (Table S1) including 25 previously known and another 15 newly designed by following the criteria.^19^ Initially, these primers were screened in a set of four different jamun genotypes and those generating polymorphic bands were selected for further study (Table 2). The polymerase chain reaction (PCR) was performed in a total volume of 20 µl reaction containing 50 ng of template DNA, 1X PCR buffer with 1.5 mM MgCl_2_ (Sigma Aldrich, India), 250 mM of each dNTPs, 0.5 µM CBDP primer and 1 U of Taq DNA polymerase (Sigma Aldrich, India). The amplification program consisted of an initial five cycles of denaturation at 94℃ for 1 min, annealing at 35℃ for 1 min and extension at 72℃ for 1 min. Following these initial cycles, the annealing temperature was then increased to 50 °C for 35 cycles, followed by a final extension at 72 °C for 10 min.

**Table 2:** Details of CBDP primers sequences and polymorphism statistics evaluated across markers.

**Primer**	**Sequence**	**TNB**	**NPB**	**PPB**	**PIC**	**MI**	**Rp**	**Band Size (bp)**	**Unique Amplicon**
CAAT-1	TGAGCACGATCCAAT AGC	12	12	100.00	0.25	2.94	7.85	450–2050	3; 1550 bp,1700 bp, 2050 bp in PCJ
CAAT-2	TGAGCACGATCCAATAAT	12	11	91.67	0.31	3.41	6.37	300–2000	−
CAAT-3	TGAGCACGATCCAAT ACC	11	11	100.00	0.28	3.11	6.24	500–2050	2; 1700 bp in PCJ-9; 20000 bp *S. fruitecosum*
CAAT-4	TGAGCACGATCCAAT AAG	15	15	100.00	0.28	4.16	9.60	480–1700	1; 2000 bp in CISH-37
CAAT-5	TGAGCACGATCCAAT CTA	8	8	100.00	0.31	2.52	4.54	300–1100	1; 1500 bp in *S. fruitecosum*
CAAT-6	TGAGCACGATCCAAT CAG	15	14	93.33	0.15	2.04	11.43	350–2500	6; 450 bp, 500 bp, 650 bp, 1400 bp, 2500 bp in *S. fruitecosum* and 950 bp in PCJ-5
CAAT-7	TGAGCACGATCCAAT CGA	16	15	93.75	0.27	4.05	9.44	350–2400	−
CAAT-9	TGAGCACGATCCAAT GAT	13	12	92.31	0.24	2.90	8.83	450–2200	1; 850 bp in PCJ-14
CAAT-10	TGAGCACGATCCAAT GTT	11	10	90.91	0.26	2.61	6.40	300–2000	1; 2000 bp in *S. fruitecosum*
CAAT-12	TGAGCACGATCCAATATA	14	13	92.86	0.28	3.69	7.99	400–2000	2; 400 bp, 500 bp in PCJ-6
CAAT-13	TGAGCACGATCCAATGAG	10	10	100.00	0.27	2.70	5.49	450–3000	1; 3000 bp in CISH-37
CAAT-14	TGAGCACGATCCAATGCG	16	16	100.00	0.34	5.50	8.48	700–3000	−
CAAT-16	TGAGCACGATCCAATTGA	14	13	92.86	0.22	2.82	10.06	600–3000	3; 1850 bp, 1900 bp in PCJ-12 and 3200 bp in PCJ-21
CAAT-17	TGAGCACGATCCAATTTG	9	9	100.00	0.23	2.07	5.97	800–2200	2; 1600 bp, 2200 bp in *S. fruitecosum*
CAAT-18	CTGAGCACGATCCAATAG	10	10	100.00	0.29	2.86	6.09	500–2000	−
CAAT-19	CTGAGCACGATCCAATAC	7	6	85.71	0.29	1.71	3.75	550–2200	1; 2500 bp in PCJ-15
CAAT-20	CTGAGCACGATCCAATAT	8	7	87.50	0.24	1.66	5.52	550–2000	−
CAAT-22	CTGAGCACGATCCAATCG	15	15	100.00	0.27	4.08	9.39	800–2200	−
CAAT-25	CTGAGCACGATCCAATGT	15	15	100.00	0.35	5.23	7.04	400–2100	3; 550 bp 800 bp in *S. fruitecosum* and 790 bp in PCJ-2
CAAT-28	TGCGTGTACACCAATTAG	9	9	100.00	0.24	2.12	6.08	400–1600	−
CAAT-35	TAGACGTGCTACCAATAC	14	14	100.00	0.24	3.29	9.33	500–2050	1; 3000 bp in PCJ-2
CAAT-36	TAGACGTGCTACCAATAT	13	13	100.00	0.24	3.18	8.70	400–1600	−
	Average	12	11	96.00	0.27	3.12	7.48	300–3000	−

***TNB***, total number of bands; ***NPB****,* number of polymorphic bands; ***PPB***, percentage of polymorphic bands; ***PIC,*** polymorphism information content; ***MI,*** marker index; ***Rp***, resolving power.

### 2.4 PCR of SCoT markers

The SCoT primers (Table S2)^20^ were synthesized from Eurofins Genomics (USA) and their sequences are provided in Table 3. PCR was set up in a reaction volume of 20 µl, containing 1X PCR buffer with 1.5 mM MgCl2 (Sigma Aldrich, India), 250 µM of each dNTP, 0.8 µM of primer, 1 U of Taq DNA polymerase (Sigma Aldrich, India) and 50 ng of DNA template. The amplification was performed in a thermocycler (Biometra Professional Thermocycler, Germany). The PCR program consisted of 35 cycles, with each cycle including denaturation at 94 °C for 1 min, annealing at 50 °C for 1 min and a 2-minute extension step. At the end, a final extension was carried out at 72℃ for 5 min to ensure complete synthesis of all PCR fragments.

**Table 3:** Details of SCoT primers sequences and polymorphism statistics evaluated across markers.

**Primer**	**Sequence**	**TNB**	**NPB**	**PPB**	**PIC**	**MI**	**Rp**	**Band Size (bp)**	**Unique Amplicon**
ScoT-1	CAACAATGGCTACCACCA	9	8	88.89	0.34	2.74	4.64	200–1400	−
ScoT-2	CAACAATGGCTACCACCC	10	8	80.00	0.30	2.39	5.62	700–3000	−
ScoT-3	CAACAATGGCTACCACCG	12	12	100.00	0.32	3.86	6.54	700–3000	−
ScoT-6	CAACAATGGCTACCACGC	9	7	77.78	0.24	1.71	5.66	450–1800	−
ScoT-12	ACGACATGGCGACCAACG	10	10	100.00	0.36	3.64	4.39	500–2500	−
ScoT-13	ACGACATGGCGACCATCG	13	13	100.00	0.32	4.19	7.21	700–3000	3; 480 bp, 600 bp in CISH-37 and 900 bp in PCJ-8
ScoT-21	ACGACATGGCGACCCACA	6	6	100.00	0.26	1.55	3.82	350–900	1; 900 bp in PCJ-16
	Average	10	9	92.00	0.31	2.87	5.41	200–3000	

***TNB***, total number of bands; ***NPB****,* number of polymorphic bands; ***PPB***, percentage of polymorphic bands; ***PIC,*** polymorphism information content; ***MI,*** marker index; ***Rp***, resolving power.

### 2.5 Electrophoresis and gel documentation

The amplified bands generated with both CBDP and SCoT markers were analysed on a 1 % agarose gel using electrophoresis in 1X TBE buffer. The amplified fragments were stained with ethidium bromide, and a standard molecular size marker (Thermo Scientific, USA) was used to accurately determine their sizes. The images of gels were recorded in a gel imaging system (Syngene, UK).

### 2.6 Statistical analysis of molecular data

The PCR gel images of CBDP and SCoT markers were visually analysed and reproducible bands were focused for scoring. A binary matrix was generated for the presence (1) and absence (0) of bands and used for various statistical and molecular analyses. To find out the effectiveness of the marker, many parameters such as polymorphic information content (PIC), marker index (MI) and resolving power (Rp) were used. PIC was computed by the following formula PIC = 2fi (1 − fi)^57^. The MI was analysed as suggested.^58^ The Rp describes a primer’s ability to detect variability between genotypes and was determined using the method. ^59^ Genetic diversity indices Nei's gene diversity index (h), the number of effective alleles (ne) and Shannon diversity index (I) were estimated using the software GenAlEx v.6.^60^ Phylogenetic trees were obtained after carrying out hierarchical clustering. This was done with the Unweighted Pair Group Method with Arithmetic Mean (UPGMA), as per Jaccard similarity coefficients. The GenAlEx v.6.5 program was used to perform PCoA and AMOVA analyses.^60^ Population structure was estimated by the STRUCTURE software program (version 2.3.4).^61^ To obtain the exact estimate of the population a total of 10 runs of STRUCTURE were performed for each k population (K = 1–10). For all runs, we set a burn-in of 10,000 iterations after which 100,000 MCMC repetitions were run. The optimal number of clusters (k) was further determined using the ΔK method.^62^ and this output was processed through a web-based platform using STRUCTURE HARVESTER v0.6.94.

## 3 Results

### 3.1 CBDP and SCoT markers polymorphism statistics

**Fig. 2:**
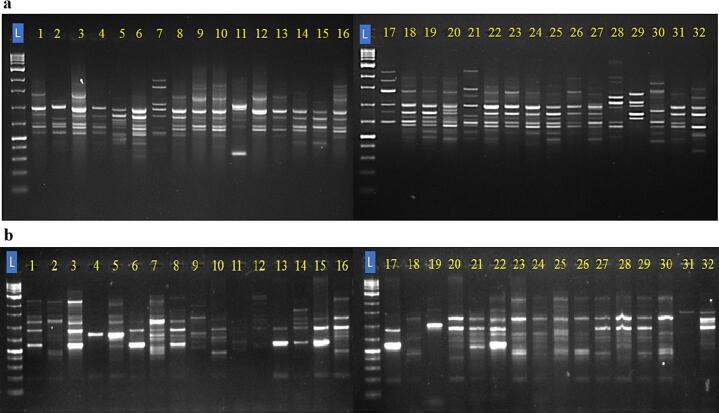
Representative gel images showing the profile of 32 jamun genotypes by using CBDP-4 (a) and SCoT-12 (b) (L: 1 kb DNA ladder).

The genetic diversity among the jamun genotypes was examined by 22 CBDP and 7 SCoT markers. The representative amplification profiles of 32 studied jamun genotypes with some CBDP and SCoT markers are presented in Fig. 2.

CBPD primers amplified a total of 291 bands out of which 276 were polymorphic and broadly revealed 94.85 % polymorphism among the studied jamun genotypes. The total number of bands (TNB) per primer ranged from 7 (CBDP-19) to 16 (CBDP-7 and CBDP-14), whereas the percentage polymorphic bands (PPB) ranged from 85.71 % for CBDP-19 to 100 % for most of the primers. The Rp values ranged from 3.75 in the case of CBDP-19 to 11.43 for CBDP-6, indicating efficiency in differentiating the genotypes by these primers. The PIC values ranged from 0.145 for CBDP-6 to 0.343 for CBDP-14, with an average PIC value of 0.277. They also reflected a mean marker index of 3.21, out of which the highest, 5.498, was contributed by CBDP-14 (Table 2).

SCoT primers generated 69 bands, out of which 64 were polymorphic, hence giving the percentage of PPB to 92.75 %. The number of bands per primer ranged from 6 (SCoT-21) to 13 (SCoT-13). PPB ranged from 77.78 % for SCoT-6 to 100 % for multiple primers like SCoT-3, SCoT-12, SCoT-13, and SCoT-21. The Rp of SCoT primers ranged from 3.82 (SCoT-21) to 7.21 (SCoT-13) with a mean Rp of 5.28, suggesting their usefulness in distinguishing jamun genotypes. The PIC values for SCoT primers ranged from 0.244 (SCoT-6) to 0.364 (SCoT-12). The marker index varied from 1.547 for SCoT-21 to 4.187 for SCoT-13 with a general mean MI value of 2.88 for all the generated markers (Table 3).

The CBDP and SCoT markers efficiently produced unique amplicons among the genotypes. *S. fruitecosum* showed unique amplicons by multiple markers, such as 2000 bp (CBDP-3), 1500 bp (CBDP-5), 2500 bp, 1400 bp, 650 bp, 500 bp, 450 bp (CBDP-6), 2000 bp (CBDP-10), 2200 bp, 1600 bp (CBDP-17), 800 bp, 550 bp (CBDP-25). These unique amplicons revealed its distinct genetic profile. CISH J-37 recorded unique bands of 2000 bp (CBDP-4) and 3000 bp (CBDP-13). Seedling-origin genotypes also showed unique amplicons, including 2050 bp, 1700 bp, 1550 bp by CBDP-1 exclusive to PCJ-9, 850 bp by CBDP-9 in PCJ-14, a unique amplicon of 3200 bp in PCJ-21 by CBDP-16 and 790 bp unique amplicon by CBDP-25, exclusive to PCJ-2 (Table 2). In the case of SCoT markers, SCoT-13 generated a unique amplicon of 900 bp in PCJ-8 and two unique amplicons (600 bp and 480 bp) in CISH J-37. Moreover, a unique amplicon of 900 bp was produced by SCoT-21 in PCJ-16 (Table 3).

### 3.2 Diversity indices of CBDP and SCoT markers

The present study showed significant genetic diversity across the CBDP and SCoT markers, as evidenced by different diversity indices (Table 4), (Table 5). Ne for CBDP markers ranged from a minimum value of 1.25 for CBDP-6 to a maximum of 1.60 for CBDP-25 with a mean value of 1.45 suggesting high genetic diversity. Gene diversity, h averaged between a minimum of 0.1457 for CBDP-6 and a maximum of 0.3484 for CBDP-25 with a mean of 0.2700. These values suggest the existence of high diversity across loci. Shannon diversity index, I varied from 0.2377 for CBDP-6 to 0.522 for CBDP-25 with an average of 0.4151, which further shows a high level of genetic diversity among loci. Among the CBDP markers, the highest genetic diversity was revealed for CBDP-25 (h = 0.3484 and I = 0.5218), whereas the lowest values were recorded for CBDP-6 (h = 0.1457 and I = 0.2377) (Table 4).

**Table 4:** Different diversity indices analysis of CBDP markers.

**Sl. No**	**Primer name**	**ne**	**h**	**I**
1.	CAAT-1	1.3988	0.2451	0.3831
2.	CAAT-2	1.5382	0.3101	0.4627
3.	CAAT-3	1.499	0.2824	0.4239
4.	CAAT-4	1.4195	0.2772	0.4409
5.	CAAT-5	1.4989	0.3144	0.4854
6.	CAAT-6	1.2504	0.1457	0.2377
7.	CAAT-7	1.4556	0.2697	0.4158
8.	CAAT-9	1.3723	0.2415	0.3836
9.	CAAT-10	1.4654	0.2611	0.3925
10.	CAAT-12	1.4819	0.2835	0.4293
11.	CAAT-13	1.4823	0.2700	0.4113
12.	CAAT-14	1.5556	0.3436	0.5220
13.	CAAT-16	1.3155	0.2169	0.3558
14.	CAAT-17	1.3714	0.2303	0.3661
15.	CAAT-18	1.4523	0.2861	0.4488
16.	CAAT-19	1.5086	0.2910	0.4365
17.	CAAT-20	1.3606	0.2373	0.3777
18.	CAAT-22	1.4243	0.2721	0.4314
19.	CAAT-25	1.6030	0.3484	0.5218
20.	CAAT-28	1.3709	0.2360	0.3786
21.	CAAT-35	1.3725	0.2353	0.3772
22.	CAAT-36	1.3778	0.2443	0.3929
23.	Average	1.44	0.27	0.41

***ne***, effective no. of alleles; ***h****,* Nei’s gene diversity; ***I****,* Shannon index.

**Table 5:** Different diversity indices analysis of SCoT markers.

**Sl. No**	**Primer name**	**ne**	**h**	**I**
1	SCoT-1	1.5729	0.3426	0.5085
2	SCoT-2	1.5194	0.2986	0.4390
3	SCoT-3	1.5262	0.3217	0.4903
4	SCoT-6	1.4175	0.2446	0.3690
5	SCoT-12	1.6411	0.3645	0.5393
6	SCoT-13	1.5200	0.3221	0.4950
7	SCoT-21	1.4289	0.2629	0.4070
	Average	1.52	0.31	0.46

***ne***, effective no. of alleles; ***h****,* Nei’s gene diversity; ***I****,* Shannon index.

For SCoT markers, Ne ranged from 1.42 (SCoT-21) to 1.64 (SCoT-12) with an average of 1.52, a little higher than the allelic diversity of the CBDP markers. Gene diversity (h) was from 0.2446 (SCoT-6) to 0.3645 (SCoT-12) and the average is 0.3081, showing greater diversity within loci. Shannon's index ranged from 0.369 in SCoT-6 to 0.539 in SCoT-12, with a mean of 0.4640, indicating more cumulative diversity in SCoT markers overall. The maximum genetic diversity was seen in the case of SCoT-12 showing h = 0.3645 and I = 0.5393. However, the lowest was found in SCoT-6 showing h = 0.2446 and I = 0.3690 (Table 5). The higher mean gene diversity and Shannon's index values of SCoT markers than CBDP indicate wider genetic variability across the loci analysed by SCoT markers.

### 3.3 Cluster analysis using CBDP, SCoT and combined markers

**Fig. 3:**
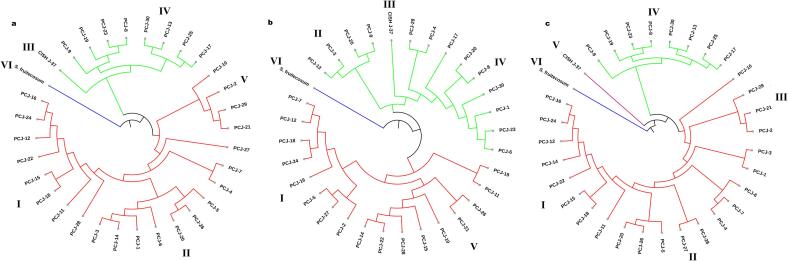
Dendrograms constructed of 32 jamun genotypes based on CBDP (a), SCoT (b) and combined CBDP + SCoT (c).

The genetic relationships among 32 jamun genotypes were evaluated using dendrograms based on CBDP, SCoT, and combined markers (Fig. 3). All analyses revealed six major clades, highlighting genetic diversity among seedling-origin, improved and wild genotypes. The UPGMA dendrogram generated using CBDP data, displayed moderate to high genetic variance with genetic distances ranging from 0.050 to 0.3692. Clade-I and Clade-II grouped most of the seedling origin genotypes like PCJ-16, PCJ-24 and PCJ-12 with distances of 0.0123 to 0.2662 that showed moderate diversity. CISH J-37 formed a separate sub-cluster in Clade-III with a genetic distance of 0.2837 showing divergence because of genetic improvement. Moreover, *S. fruitecosum* was expected placed as a genetic outlier in the Clade-VI, having the maximum genetic distance of 0.3692.

Similarly, the UPGMA dendrogram constructed with SCoT data showed genetic distance among jamun genotypes from 0.050 to 0.2924, which is similar to CBDP findings. Like CBDP-based clustering, Clade-I in this dendrogram started with seedling-origin genotypes, PCJ-16, PCJ-24 and PCJ-12 clustered at a distance of 0.0125–0.1866 while PCJ-23 and PCJ-8 got placed in Clade-IV with a very low divergence of 0.1429. Clade-VI was assigned to *S. fruitecosum*, whose wild genotype status was further explained by a maximum genetic distance of 0.2924, while CISH J-37 stayed in Clade-III with a genetic distance of 0.2510.

The combined-set analysis established six clades. Clade-I shows genotypes of seedling origin clustered between 0.1728 and 0.2074 and Clade-IV included PCJ-23 and PCJ-8 at 0.1467 genetic distance. On the other hand, the improved cultivar (CISH J-37), was genetically separated from genotypes of seedling origin by 0.2981 and mapped to Clade-V. *S. fruitecosum* emerged as the outlier in Clade-VI, showing a genetic distance of 0.3520.

### 3.4 Principal coordinates analysis (PCoA) and analysis of molecular variance (AMOVA)

**Fig. 4:**
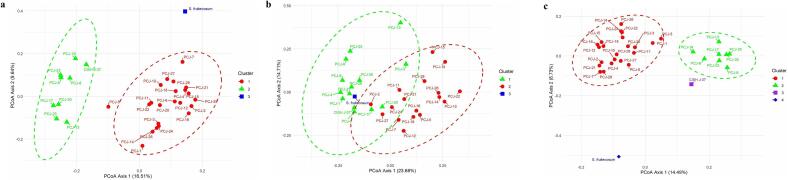
Principal coordinates analysis (PCoA) based on CBDP (a), SCoT (b) and combined CBDP + SCoT (c).

Principal Coordinates Analysis (PCoA) was separately performed for CBDP, SCoT and combined both markers data which explained genetic variation of 26.65 %, 38.39 % and 23.22 %, respectively (Fig. 4). PCoA analysis based on CBDP marker data revealed three clusters. The first cluster contained closely related seedling-origin genotypes, CISH J-37 whereas, *S. fruitecosum* formed different clusters for the CBDP marker. There was a considerable genetic difference among the seedling-origin genotypes, CISH J-37 and *S. fruitecosum*. Additionally, three groups in the SCoT study display genotypes of seedling origin in cluster 2 (PCJ-19, PCJ-22, and PCJ-28) and cluster 1 (PCJ-6, PCJ-23, and PCJ-13), with minimal intermixing. *S. fruitecosum*, though, remained alone. The proximity of CISH J-37 to *S. fruitecosum* reveals its divergence from the seedling genotypes. Furthermore, the combined analysis displayed four clusters in which CISH J-37 constituted a distinct cluster. The genotypes of seedling origin separated into two clusters, alongside *S. fruitecosum* remaining an outlier in cluster 4 due to its extreme genetic variation.

**Fig. 5:**
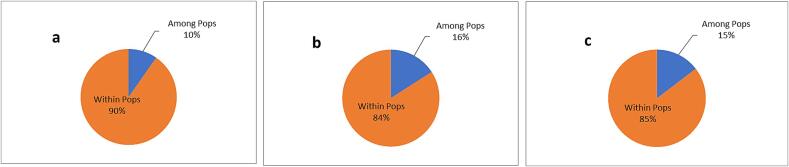
Molecular Variance Analysis using CBDP, SCoT and combined CBDP + SCoT.

Analysis of Molecular Variance (AMOVA) for CBDP markers showed a 16 % variation among populations (Table 6). At the same time, 84 % of the total variation is seen within populations. In contrast, SCoT markers revealed that there was 90 % molecular variation within the population and 10 % molecular variation among the population. Further, combined analysis of both CBDP and SCoT markers revealed 15 % of the variation among the population and 85 % within the population (Fig. 5).

**Table 6:** AMOVA results based on CBDP, SCoT and combined (CBDP + SCoT) markers.

**Source of variation**	**df**	**SS**	**MS**	**Est. Var**	**% Var**
**CBDP**					
**Among Pops**	1	68.406	68.406	7.571	16 %
**Within Pops**	30	1200.500	40.017	40.017	84 %
**Total**	31	1268.906		47.587	100 %
**SCoT**					
**Among Pops**	1	15.758	15.758	1.208	10 %
**Within Pops**	30	336.867	11.229	11.229	90 %
**Total**	31	352.625		12.437	100 %
**CBDP + SCoT**					
**Among Pops**	1	84.117	84.117	8.768	15 %
**Within Pops**	30	1537.133	51.238	51.238	85 %
**Total**	31	1621.250		60.005	100 %

***Df****,* degrees of freedom, ***SS*** sum of square, ***MS*** mean sum of square, ***% Var*** percentage variance.

### 3.5 Population structure analysis

**Fig. 6:**
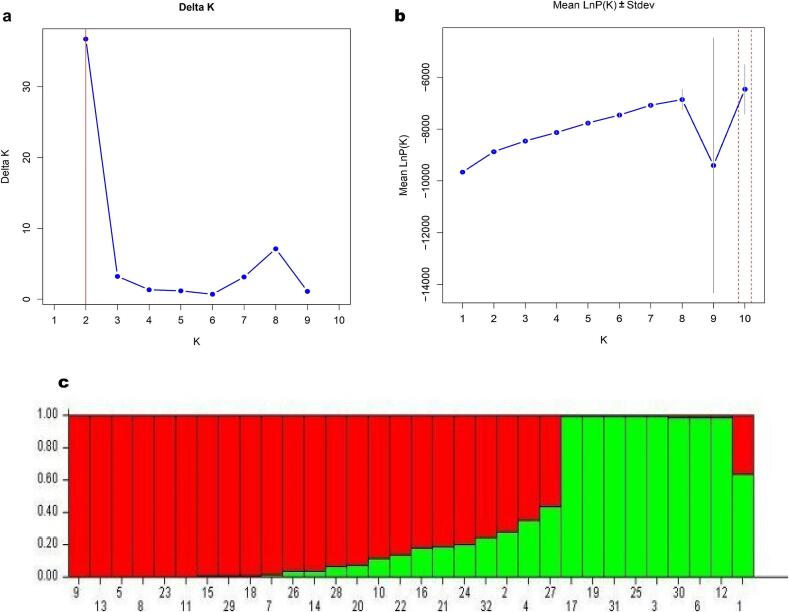
Population structure analysis based on combined CBDP + SCoT markers. Delta K analysis (a), Mean LnP (K) plot (b), the admixture plot (c).

The population structure of the 32 jamun genotypes was assessed using a combined dataset of CBDP and SCoT markers. Delta K analysis led to an optimal K value of 2. Therefore, the genotypes were divided into two major populations. Consistent with the two major genetic groups, the Mean LnP (K) plot for those corresponds to a very low variation in likelihood for K over two. Again, two distinct populations were seen in the admixture plot (Fig. 6). Here, the first population was mostly represented by seedling-origin genotypes like, PCJ-16, PCJ-24 and PCJ-12. These genotypes showed lower levels of admixture and thus pointed to a significant degree of genetic homogeneity in this population. On the other hand, CISH J-37, which was differentiated from *S. fruitecosum,* appeared in the second graph in green. It showed more important levels of genetic admixture. This graph shows that even though CISH J-37 was an improved variety, it still has some genetic relationship with wild populations. This is seen in the small amount of mixing between the groups in the plot. The population structure analysis shows the population structure of different seedling-origin genotypes and the wild/improved genotypes revealing genetic divergence in the original lot. Major two populations are genetically well differentiated, with minimum gene flow between them.

## 4 Discussion

The *Syzygium* genus encompasses a vast number of species, including several cultivable ones such as Indian blackberry (*Syzygium cumini*), Java apple (*Syzygium aromaticum*) and Malay apple (*Syzygium malaccense*). Among these, jamun (*Syzygium cumini*) is native to India, and thus germplasm collection of this crop from India may represent a valuable asset for its genetic improvement and breeding programs. Therefore, a detailed understanding of the extent and patterns of diversity in the jamun gene pool of India is crucial for their effective conservation and utilization in genetic improvement programs. In the past two decades, gene-based molecular markers have proved very useful for characterizing and understanding genetic relationships among germplasm collections of crops.[40], [55] To our knowledge, this study for the first time reports characterization of diversity among jamun genotypes employing two different gene-based marker techniques, CBDP and SCoT.

The present study analysed genetic diversity among the 32 jamun genotypes using 22 CBDP and 7 SCoT primers. The percentage polymorphism (94.85 %) for CBDP markers observed in this study was higher as compared to an earlier study using the same marker.^55^ The SCoT markers also exhibited a similar level of polymorphism (92.75 %) which was comparable to the results of the previous report.^26^ Both gene-targeted markers showed higher polymorphism percentages than previous studies on conventional markers, RAPD, reported polymorphism ranging from 41.2 % to 87.50 %.[46], [47], [52], [53] ISSR demonstrated polymorphism 33.3 % to 64.2 %.[49], [51], [52], [53]

PIC and MI values are considered very useful marker statistics parameters for characterizing genetic diversity in crops. The averages of the PIC and MI parameters for the SCoT marker were significantly higher than CBDP markers, which was similar to the findings^63^ However, the PIC value of both these markers was higher than ISSR (0.248), while SCoT markers represented a higher PIC value than RAPD (0.285) markers as reported in the previous study.^53^ Rp values for the CBDP marker ranged from 3.75 to 11.43, while for the SCoT, they varied from 3.82 to 7.21, showing that these primers were effective in distinguishing the genotypes. The Rp values for markers recorded in this study are greater than earlier study.^55^ Higher Rp and MI values revealed that these primers possess greater potency and better resolution.^65^ Consistent with our findings, earlier research has also documented the distinctive performance of SCoT and CBDP markers in analysing genetic diversity and molecular fingerprinting in different plant species.[35], [63], [64], [65] Genotype-specific amplicons demonstrate the polymorphism capability of CBDP and SCoT markers and could be useful for genotype identification and genetic diversity studies. Moreover, *S. fruitecosum* was included as an outgroup in our study and showed unique amplicons by multiple markers.

The genetic diversity index (*h*) quantifies the genetic variation within a population, thus indicating overall genetic diversity and differentiation across genotypes. In comparison, Shannon's index (*I*) measures both the richness and evenness of genetic features between and within populations.^66^ Higher values of the genetic diversity index (*h*) and Shannon's index (*I*) indicate a greater level of genetic diversity within and among populations. The *h* and *I* indices obtained in the current study revealed high diversity among loci and findings aligned with many studies.[44], [55] Investigations have demonstrated that markers such as SCoT and CBDP provide greater *h* and *I* value, highlighting their efficacy in capturing genetic heterogeneity among various species, including populations of mango and wheat.[40], [67]

The genetic relationship among jamun genotypes was demonstrated by clustering and PCoA analyses. Cluster analysis is one of the most effective methods for analyzing phylogenetic relationships and classifying genetic groups.^66^ The dendrograms for all 32 genotypes were created using the UPGMA approach. The cluster analysis of CBDP and SCoT markers identified six sub-clusters in which the majority of the genotypes originating from seedling origin, such as PCJ-16, PCJ-24 and PCJ-12, exhibiting moderate diversity, were grouped into Clades I and II. The results obtained in our study were consistent with earlier reports.[19], [44] *S. fruitecosum* was classified as a genetic outlier by both the markers, while CISH J-37 established a separate sub-cluster showing divergence due to genetic improvement. Similar observations were noted in *Andrographis paniculata*.^34^ A dendrogram produced with cumulative CBDP and SCoT data yielded many subclusters, similar to results demonstrated in *Prosopis cineraria.*^35^

PCoA effectively illustrated distinct clustering patterns demonstrating the genetic differentiation among species and populations based on the polymorphic fragments generated by CBDP and SCoT markers. PCoA-based clustering of jamun genotypes largely concurred with the findings of UPGMA analysis. The total genetic variation explained by CBDP, SCoT and combined markers through PCoA analysis was 26.65 %, 38.39 % and 23.22 % and in line with the findings, where they revealed 33 % variation in the top three coordinates for PCoA analysis of SCoT-based markers.^34^ Also, 20.90 % total variation was observed when both markers were combined.^35^ The PCoA plot was constructed using the CBDP data and grouped all studied jamun genotypes in three clusters. Cluster 1 contained closely related seedling-origin genotypes. SCoT markers also formed three clusters with little intermixing among genotypes. CISH J-37 and *S. fruitecosum* formed different clusters for both the markers as well as for combined data, showing higher genetic variations. Our study shows significant genetic variations among wild, improved variety and seedling-origin accessions and the results on similar lines were also observed.^44^ Several studies revealed a higher percentage of variation within the population than among the population, similar to our results, indicating a significant genetic difference across all accessions within the population.[68], [69], [70], [39] demonstrated that CBDP and SCoT data individually showed larger genetic diversity within the population as compared to between populations.^35^

Utilizing a combined dataset of SCoT and CBDP markers, the population structure of the 32 jamun genotypes was analysed. Population structure-based grouping of jamun genotypes was broadly on the lines obtained using UPGMA and PCoA methods. Many earlier investigations have also demonstrated the utility of gene-based markers in efficiently delineating genetic diversity in crops according to their genomic backgrounds.[71], [72]

## 5 Conclusion

This study represents a novel attempt to combine CBDP and SCoT markers, enabling a comprehensive genetic diversity assessment across Indian blackberry genotypes. The study evaluated the effectiveness of these gene-based markers in revealing polymorphism, genetic relationships and population structure among seedling-origin, improved and wild genotypes. The results highlighted the greater efficiency of CBDP markers in identifying polymorphisms and discriminating genotypes than SCoT markers. Genotype-specific unique amplicons identified in this research may be useful for genotype identification and diversity studies. Moreover, the combined application of CBDP and SCoT markers proved to be a reliable approach for characterizing genetic diversity in jamun and providing valuable insights into its genomic resources. The genetic diversity revealed in this study can be helpful for the conservation and management of genetic resources of Indian blackberry. Furthermore, it is expected to motivate researchers to explore the genetic diversity of this kind of medicinally important and underutilized fruit crops using reliable molecular markers.

## CRediT authorship contribution statement

**Ajay Kumar:** Writing – original draft, Validation, Methodology, Investigation, Formal analysis, Data curation. **Kanhaiya Singh:** Writing – review & editing, Supervision, Project administration, Conceptualization. **Amit Kumar Singh:** Writing – review & editing, Resources, Project administration, Conceptualization. **Jai Prakash:** Writing – review & editing, Resources, Conceptualization. **Amit Kumar Goswami:** Writing – review & editing, Resources, Project administration, Conceptualization. **Gyan Prakash Mishra:** Writing – review & editing, Resources, Methodology. **Vishaw Bandhu Patel:** Writing – review & editing, Resources, Investigation, Conceptualization. **Suman Lata:** Writing – review & editing, Resources. **Anshuman Singh:** Writing – review & editing, Resources.

## Declaration of competing interest

The authors declare that they have no known competing financial interests or personal relationships that could have appeared to influence the work reported in this paper.
