# Supplementary material for: Genetic diversity and population structure analysis of Indian blackberry (Syzygium cumini L.) using CAAT box‑derived polymorphism (CBDP) and start codon targeted polymorphism (SCoT) markers
Source: J Genet Eng Biotechnol. 2025 Feb 13;23(1):100468. doi: 10.1016/j.jgeb.2025.100468 (PMC11875165; doi:10.1016/j.jgeb.2025.100468)
Supplement: Supplementary Data 1 [file mmc1.docx]

Table S1 CBDP primer sequences used in this study

| **Primer name** | **Primer sequence** | **GC %** |
| --- | --- | --- |
| CAAT- 1 | TGAGCACGATCCAAT AGC | 50.0 |
| CAAT -2 | TGAGCACGATCCAATAAT | 39.0 |
| CAAT -3 | TGAGCACGATCCAAT ACC | 50.0 |
| CAAT -4 | TGAGCACGATCCAAT AAG | 44.4 |
| CAAT -5 | TGAGCACGATCCAAT CTA | 44.4 |
| CAAT -6 | TGAGCACGATCCAAT CAG | 50.0 |
| CAAT -7 | TGAGCACGATCCAAT CGA | 50.0 |
| CAAT -8 | TGAGCACGATCCAAT CGG | 55.6 |
| CAAT -9 | TGAGCACGATCCAAT GAT | 44.4 |
| CAAT -10 | TGAGCACGATCCAAT GTT | 44.4 |
| CAAT -11 | TGAGCACGATCCAAT TGC | 50.0 |
| CBDP-12 | TGAGCACGATCCAATATA | 39.0 |
| CAAT -13 | TGAGCACGATCCAATGAG | 50.0 |
| CAAT -14 | TGAGCACGATCCAATGCG | 55.6 |
| CAAT -15 | TGAGCACGATCCAATTGA | 44.4 |
| CAAT -16 | TGAGCACGATCCAATTGA | 44.4 |
| CAAT -17 | TGAGCACGATCCAATTTG | 44.4 |
| CAAT -18 | CTGAGCACGATCCAATAG | 50.0 |
| CAAT -19 | CTGAGCACGATCCAATAC | 50.0 |
| CAAT -20 | CTGAGCACGATCCAATAT | 44.4 |
| CAAT -21 | CTGAGCACGATCCAATCA | 50.0 |
| CAAT -22 | CTGAGCACGATCCAATCG | 55.6 |
| CAAT -23 | CTGAGCACGATCCAATGG | 55.6 |
| CAAT -24 | CTGAGCACGATCCAATGA | 50.0 |
| CAAT -25 | CTGAGCACGATCCAATGT | 50.0 |
| CAAT-26 | TGCGTGTATACCAATAGA | 39.0 |
| CAAT-27 | TGCGTGTATACCAATACG | 44.4 |
| CAAT-28 | TGCGTGTACACCAATTAG | 44.4 |
| CAAT-29 | TGCGTGTACACCAATGGA | 50.0 |
| CAAT-30 | TTGCGTGTACACCAATCG | 50.0 |
| CAAT-31 | TTGCGTGTACACCAATGC | 50.0 |
| CAAT-31 | TTGCGTGTACACCAATAG | 44.4 |
| CAAT-33 | TTGCGTGTACACCAATTG | 44.4 |
| CAAT-34 | TAGACGTGCTACCAATAG | 44.4 |
| CAAT-35 | TAGACGTGCTACCAATAC | 44.4 |
| CAAT-36 | TAGACGTGCTACCAATAT | 39.0 |
| CAAT-37 | TAGACGTGCTACCAATCA | 44.4 |
| CAAT-38 | TAGACGTGCTACCAATCG | 50.0 |
| CAAT-39 | TAGACGTGCTACCAATGC | 50.0 |
| CAAT-40 | TAGACGTGCTACCAATAG | 44.4 |
| CAAT-41 | TAGACGTGCTACCAATGT | 44.4 |

**Table S2** SCoT primer sequences used in this study

| **Primer name** | **Primer sequence** | **GC %** |
| --- | --- | --- |
| SCoT-1 | CAACAATGGCTACCACCA | 50 |
| SCoT-2 | CAACAATGGCTACCACCC | 56 |
| SCoT-3 | CAACAATGGCTACCACCG | 56 |
| SCoT-4 | CAACAATGGCTACCACCT | 50 |
| SCoT-5 | CAACAATGGCTACCACGA | 50 |
| SCoT-6 | CAACAATGGCTACCACGC | 56 |
| SCoT-7 | CAACAATGGCTACCACGG | 56 |
| SCoT-8 | CAACAATGGCTACCACGT | 50 |
| SCoT-9 | CAACAATGGCTACCAGCA | 50 |
| SCoT-10 | CAACAATGGCTACCAGCC | 56 |
| SCoT-11 | AAGCAATGGCTACCACCA | 50 |
| SCoT-12 | ACGACATGGCGACCAACG | 61 |
| SCoT-13 | ACGACATGGCGACCATCG | 61 |
| SCoT-14 | ACGACATGGCGACCACGC | 67 |
| SCoT-15 | ACGACATGGCGACCGCGA | 67 |
| SCoT-16 | ACCATGGCTACCACCGAC | 56 |
| SCoT-17 | ACCATGGCTACCACCGAG | 61 |
| SCoT-18 | ACCATGGCTACCACCGCC | 67 |
| SCoT-19 | ACCATGGCTACCACCGGC | 67 |
| SCoT-20 | ACCATGGCTACCACCGCG | 67 |
| SCoT-21 | ACGACATGGCGACCCACA | 61 |
| SCoT-22 | AACCATGGCTACCACCAC | 56 |
| SCoT-23 | CACCATGGCTACCACCAG | 61 |
| SCoT-24 | CACCATGGCTACCACCAT | 56 |
| SCoT-25 | ACCATGGCTACCACCGGG | 67 |
| SCoT-26 | ACCATGGCTACCACCGTC | 61 |
| SCoT-27 | ACCATGGCTACCACCGTG | 61 |
| SCoT-28 | CCATGGCTACCACCGCCA | 67 |
| SCoT-29 | CCATGGCTACCACCGGCC | 72 |
| SCoT-30 | CCATGGCTACCACCGGCG | 72 |
| SCoT-31 | CCATGGCTACCACCGCCT | 67 |
| SCoT-32 | CCATGGCTACCACCGCAC | 67 |
| SCoT-33 | CCATGGCTACCACCGCAG | 67 |
| SCoT-34 | ACCATGGCTACCACCGCA | 61 |
| SCoT-35 | CATGGCTACCACCGGCCC | 72 |
| SCoT-36 | GCAACAATGGCTACCACC | 56 |
